# Cancer risk in close relatives of women with early-onset breast cancer – a population-based incidence study

**DOI:** 10.1038/sj.bjc.6690106

**Published:** 1999-02

**Authors:** J H Olsen, N Seersholm, J D Boice Jr, S Krüger Kjær, J F Fraumeni Jr

**Affiliations:** Institute of Cancer Epidemiology, Danish Cancer Society, Strandboulevarden 49, Copenhagen, DK-2100, Denmark; International Epidemiology Institute, 1550 Research Boulevard, 2nd Floor, Rockville, MD, 20850, USA; Division of Cancer Epidemiology and Genetics, National Cancer Institute, Executive Plaza North 543, 6130 Executive Boulevard, Bethesda, MD, 20892, USA

**Keywords:** early-onset breast cancer, familial cancer, heriditary cancer syndromes, cancer susceptibility genes

## Abstract

Inherited susceptibility to breast cancer is associated with an early onset and bilateral disease. The extent of familial risks has not, however, been fully assessed in population-based incidence studies. The purpose of the study was to quantify the risks for cancers of the breast, ovary and other sites of close relatives of women in whom breast cancer was diagnosed at an early age. Records collected between 1943 and 1990 at the Danish Cancer Registry were searched, and 2860 women were found in whom breast cancer was diagnosed before age 40. Population registers and parish records were used to identify 14 973 parents, siblings and offspring of these women. Cancer occurrence through to 31 December 1993 was determined within the Cancer Registry's files and compared with national incidence rates. Women with early-onset breast cancer were at a nearly fourfold increased risk of developing a new cancer later in life (268 observed vs 68.9 expected). The excess risk was most evident for second cancer of the breast (181 vs 24.5) and for ovarian cancer (20 vs 3.3). For mothers and sisters, risks for cancers of the breast and ovary were significantly increased by two- to threefold. Bilateral breast cancer and breast–ovarian cancer were very strong predictors of familial risks, with one in four female relatives predicted to develop breast and/or ovarian cancer by age 75. Mothers had a slightly increased risk of colon cancer, but not endometrial cancer. The risk for breast cancer was also increased among fathers (standardized incidence ratio 2.5; 95% CI 0.5–7.4) and especially brothers (29; 7.7–74), although based on small numbers. The risk for prostatic cancer was unremarkable. In this large population-based survey, the first-degree relatives of women who developed breast cancer before age 40 were prone to ovarian cancer as well as male and female breast cancer, but not other tumours that may share susceptibility genes with breast cancer. © 1999 Cancer Research Campaign


*British Journal of Cancer* (1999) **79**(3/4), 673–679
© 1999 Cancer Research Campaign
Article no. bjoc.1998.0106

### Cancer risk in close relatives of women with early-onset breast cancer – a population-based incidence study

**JH Olsen[1], N Seersholm[1], JD Boice Jr[2,3], S Krüger Kjær[1] and JF Fraumeni Jr[3]**

[1]Institute of Cancer Epidemiology, Danish Cancer Society, Strandboulevarden 49, DK-2100 Copenhagen, Denmark; [2]International Epidemiology Institute, 1550 Research Boulevard, 2nd Floor, Rockville, MD 20850, USA; [3]National Cancer Institute, Division of Cancer Epidemiology and Genetics, Executive Plaza North 543, 6130 Executive Boulevard, Bethesda, MD 20892, USA

**Summary** Inherited susceptibility to breast cancer is associated with an early onset and bilateral disease. The extent of familial risks has not, however, been fully assessed in population-based incidence studies. The purpose of the study was to quantify the risks for cancers of the breast, ovary and other sites of close relatives of women in whom breast cancer was diagnosed at an early age. Records collected between 1943 and 1990 at the Danish Cancer Registry were searched, and 2860 women were found in whom breast cancer was diagnosed before age 40. Population registers and parish records were used to identify 14 973 parents, siblings and offspring of these women. Cancer occurrence through to 31 December 1993 was determined within the Cancer Registry's files and compared with national incidence rates. Women with early-onset breast cancer were at a nearly fourfold increased risk of developing a new cancer later in life (268 observed vs 68.9 expected). The excess risk was most evident for second cancer of the breast (181 vs 24.5) and for ovarian cancer (20 vs 3.3). For mothers and sisters, risks for cancers of the breast and ovary were significantly increased by two- to threefold. Bilateral breast cancer and breast–ovarian cancer were very strong predictors of familial risks, with one in four female relatives predicted to develop breast and/or ovarian cancer by age 75. Mothers had a slightly increased risk of colon cancer, but not endometrial cancer. The risk for breast cancer was also increased among fathers (standardized incidence ratio 2.5; 95% CI 0.5–7.4) and especially brothers (29; 7.7–74), although based on small numbers. The risk for prostatic cancer was unremarkable. In this large population-based survey, the first-degree relatives of women who developed breast cancer before age 40 were prone to ovarian cancer as well as male and female breast cancer, but not other tumours that may share susceptibility genes with breast cancer.

**Keywords:** early-onset breast cancer; familial cancer; heriditary cancer syndromes; cancer susceptibility genes

Breast cancer is the commonest cancer among women in the Western world, with the cumulative lifetime risk in Denmark being 1 in 12 (Danish Board of Health, 1997). Together with ovarian cancer, breast cancer constitutes almost one-third of all incident cancers among women (non-melanoma skin cancer excluded). The association of elevated risks for breast and ovarian cancers with a family history of these malignancies is well established (Amos and Struewing, 1993; Eby et al, 1994; Newman et al, 1997; Pharoah et al, 1997). The overall likelihood that a first-degree female relative of a breast cancer patient will develop breast or ovarian cancer is estimated to be two to three and three to four times higher, respectively, than that of other women. In many of these families, genetic determinants play a major role. It has been estimated that approximately 3% of all breast cancers and 4–5% of ovarian cancers occur among the 0.1–0.3% of women in the general population who carry germline mutations of the *BRCA1* susceptibility gene (Claus et al, 1991; Ford et al, 1995; Whittemore et al, 1997). Among women with breast cancer before the age of 35, the gene carrier prevalence is even higher, probably about 7–13% (FitzGerald et al, 1996; Langston et al, 1996), although recent population-based studies of young affected women without a positive family history suggest prevalences that are of the order of 6% (Malone et al, 1998). The *BRCA1* gene seems to have a high lifetime penetrance: approximately 55–75% for the breast cancer phenotype and 15–30% for the ovarian cancer phenotype (Struewing et al, 1997; Whittemore et al, 1997). The penetrance estimates from broader population studies tend to be lower than the estimates from families selected for linkage. In addition, familial breast cancer, especially at early ages, has been linked to germline mutations of the *BRCA2* gene as well as the *p53* gene in Li–Fraumeni syndrome and the *PTEN* gene in Cowden's disease (Gudmundsson et al, 1996; Rebbeck et al, 1996; Crook et al, 1997; Krainer et al, 1997 Li et al, 1997). The *ATM* gene also has been suspected to play a role in breast cancer occurrence (Bebb et al, 1997), although the evidence is not convincing for early-onset breast cancer (FitzGerald et al, 1997).

A familial tendency to breast cancer has been reported in association with other cancers, including those of the endometrium, prostate and colon (Tulinius et al, 1992*a*; Goldar et al, 1994; Sellers et al, 1994), but the evidence is inconclusive and has not been fully assessed in population-based incidence studies. Among some populations, the risk of prostate cancer is increased in *BRCA1* and *BRCA2* carriers (Struewing et al, 1997).

### PATIENTS AND METHODS

A population-based study of breast cancer in Denmark was conducted to quantify the risk for a second primary cancer among early-onset breast cancer patients and to investigate the risks for breast and other cancers among first-degree relatives.

*Received 17 April 1998*
*Accepted 1 June 1998*

*Correspondence to:* JH Olsen

### Study population

During 1943–90, breast cancer was diagnosed in 2860 women born in 1935 or later before they reached 40 years of age. These early-onset breast cancer patients were identified from the files of the Danish Cancer Registry, which also provided the patients' names, dates of birth and unique personal identification numbers. The last, which incorporates sex and date of birth and permits accurate linkage of information between registers, was available for 2850 women alive on 1 April 1968, when the Central Population Register (CPR) was established in Denmark and for one woman born after that date. Linkage to the files of the CPR provided information on the date of death or date of emigration. For nine women who died before 1 April 1968, the date of death was obtained manually from the Death Certificate File at the National Board of Health. Nearly 40% of the women studied were under the age of 35 when their breast cancer was diagnosed (Table 1).

### Identifying relatives

Parents of these women were identified in one of two ways. When the personal identification number was available, computerized linkage with the files of the CPR readily identified the name, identification number and date of death or emigration (if applicable) of each parent. If the breast cancer patients or one or both of the parents had died before 1 April 1968, information about the parents had to be obtained manually from 'parish registries', that is the population registers of the localities in which the families had lived at the date of birth of the cancer patient. Parents identified in this manner were then traced by computer in the CPR (parents of the nine breast cancer patients who died before 1 April 1968) or traced manually in the Death Certificate File (parents deceased before 1 April 1968) to verify personal data and dates of death. This parental search revealed that 20 pairs of the young breast cancer patients were sisters. Overall, 2670 different mothers (94%) and 2587 different fathers (91%) were found (Table 2).

Sisters and brothers of the women with early-onset breast cancer were also located in the files of the CPR by using the personal identification number of the mother or from local parish registers. For the 20 pairs of sisters with early-onset breast cancer, the sister who was youngest at the time of diagnosis was regarded as the proband of the family. A total of 2156 sisters and 2354 brothers (of 2840 probands) were identified. The offspring of the young breast cancer patients were located through the CPR from the personal identification number of their mother. A total of 2544 daughters and 2662 sons (likewise of 2840 probands) were identified.

**Table 1** Selected characteristics of the 2860 women with early-onset breast cancer in Denmark, 1943–90

| Characteristic | Number | % |
|---|---|---|
| Breast cancer patients | 2860 | 100 |
| Year of birth | | |
| 1935–44 | 1194 | 42 |
| 1945–54 | 1453 | 51 |
| 1955–68 | 213 | 7 |
| Year of diagnosis | | |
| 1953–62 | 7 | <1 |
| 1963–72 | 248 | 9 |
| 1973–82 | 1321 | 46 |
| 1983–90 | 1284 | 45 |
| Age at diagnosis | | |
| 10–14 | 1 | <1 |
| 15–19 | 2 | <1 |
| 20–24 | 39 | 1 |
| 25–29 | 271 | 10 |
| 30–34 | 807 | 28 |
| 35–39 | 1740 | 61 |
| Vital status as of 31 December 1993 | | |
| Alive | 1581 | 55 |
| Dead | 1270 | 44 |
| Emigrated | 9 | <1 |

[a] Includes 20 pairs of sisters with early-onset breast cancer.

### Identifying cancer in patients and relatives

Data on the women with early-onset breast cancer and their relatives were linked with the Danish Cancer Registry, using the study subjects' personal identification numbers or, if they had died before 1 April 1968, their date of birth, date of death and name. The period of follow-up for the occurrence of cancer among women with early-onset breast cancer extended from the date of the initial breast cancer to the date of death or emigration or until 31 December 1993, whichever came first. The period of follow-up for the occurrence of cancer among parents extended from the date of birth of the proband with breast cancer, and that among siblings and offspring from the date of birth to the date of death or emigration to 31 December 1993. Cancers, including benign tumours of

**Table 2** Year of birth and vital status for 14 973 first-degree relatives of 2840 probands with early-onset breast cancer

| Relationship | Number located | Year of birth (range) | Vital status[a] | | | | | |
|---|---|---|---|---|---|---|---|---|
| | | | Alive | | Dead | | Emigrated | |
| | | | No. | % | No. | % | No. | % |
| Mothers | 2670 | 1890–1949 | 1602 | 60 | 1061 | 40 | 7 | <1 |
| Fathers | 2587 | 1865–1949 | 1007 | 39 | 1569 | 61 | 11 | <1 |
| Sisters[b] | 2156 | 1910–1979 | 1932 | 90 | 177 | 8 | 47 | 2 |
| Brothers | 2354 | 1910–1974 | 2094 | 89 | 206 | 9 | 54 | 2 |
| Daughters | 2544 | 1950–1993 | 2456 | 96 | 22 | 1 | 66 | 3 |
| Sons | 2662 | 1950–1993 | 2563 | 96 | 60 | 2 | 39 | 2 |
| All relatives | 14973 | 1890–1993 | 11654 | 78 | 3095 | 21 | 224 | 1 |

[a]As of 31 December 1993. [b]Includes 20 sisters with early-onset breast cancer.

 © Cancer Research Campaign 1999

the brain and papillomas of the urinary tract, were classified according to the modified Danish verion of the International Classification of Diseases, 7th Revision (ICD-7) (World Health Organization, 1957). The national incidence rates for these categories of tumour, calculated according to sex, age (in 5- year groups), and 5- year historical periods, were applied to the person–years of observation for each study cohort to obtain the number of cancers that would have been expected if they had had the same rates of incidence as the general population.

### Statistical analysis

The statistical methods were chosen on the basis of the assumption that the observed number of cases of cancer in any specific category follows a Poisson distribution. Significance and confidence intervals for the standardized incidence ratio (SIR) – the ratio of the observed to the expected number of cancers – were calculated, with use of the Miettinen exact confidence limits if the observed number of cases was small; otherwise, an accurate asymptotic approximation was used (Rothman and Boice, 1979). Account was also taken of the age of the proband at the time of the initial diagnosis of breast cancer and the age of the relatives at the time of cancer diagnosis. Separate analyses were performed for relatives of probands with contralateral breast cancer ($n$ = 201) and probands with a second primary cancer of the ovary ($n$ = 20).

### RESULTS

### Second malignant neoplasms

Breast cancer was diagnosed in almost 60 women per year before the age of 40 during the years 1943–90 in Denmark. A total of 2860 patients accrued 23 000 person–years of follow-up (average 8.0 years; range >0–39.5 years), during which time 268 new primary cancers developed, with 68.9 expected, yielding a SIR of 3.9 (95% CI 3.4–4.4) (Table 3) and a cumulative risk after 25-years of follow-up of 31%. The excess 200 cancers consisted mainly of tumours of the contralateral breast (SIR 7.4; $n$ 181) and ovarian cancer (SIR 6.0; $n$ 20). A small but significant increase in risk was seen for cancers at all other sites combined (SIR 1.6). The risk for cancer of the endometrium was elevated (SIR 2.7) but not significantly. Cancer of the lung was significantly elevated (SIR 2.7), as was the risk of cancer of the liver though based on only two cases. Thyroid cancer was increased but not significantly, based on only two cases. Twenty-one women with synchronous bilateral cancer were not included in the risk estimates for second primary breast cancers.

### Female relatives

The 2670 mothers of women with early-onset breast cancer represented nearly 110 000 person–years of follow-up (average 41 years; range >0–51 years). The 2156 sisters represented some 93 000 person–years (average 43 years; range >0–51 years). Over the entire follow-up period, 889 malignancies were diagnosed in mothers and 196 in sisters, yielding SIRs of 1.3 (95% CI 1.2–1.4) and 1.6 (1.4–1.8) respectively (Table 4). Significant excess risks were seen for cancers of the breast (mothers SIR 2.0; sisters 2.6), ovary (mothers 1.7; sisters 1.9), and other sites combined (mothers 1.1). The mothers also had significantly increased risks for cancers of the cervix (SIR 1.4) and colon (SIR 1.2), whereas the SIR for cancer of the brain (0.6) was below that expected. No other significant deviations were seen among mothers or sisters, although seven cases of leukaemia were observed among sisters with 3.0 expected.

The cumulative risk for breast or ovarian cancer in the mothers was 3.8% vs 1.5% expected at the 20-year follow-up point and 12.1% v 6.2% expected at 40 years. The cumulative risk in sisters was 1.7% v 0.6% at 40 years.

A total of 2544 daughters of women with early-onset breast cancer were identified, representing approximately 56 000 person–years of follow-up. Their average age in 1993, however, was only 21.6 years. Nine cancers were observed compared with 10.7 expected, yielding a SIR of 0.8 (95% CI 0.4–1.6). Of these, two were breast cancer, diagnosed at the ages of 26 and 31, compared with 0.4 expected (SIR 5.0; 95% CI 0.4–18). Two cases of leukaemia (1.7 expected) and one case of brain cancer (2.3 expected) occurred in daughters, whereas no bone or connective tissue sarcomas were seen (data not shown).

The relative risks for cancers of the breast (Table 5) and ovary (Table 6) among first-degree female relatives were inversely related to the age at diagnosis of early-onset breast cancer in the proband. That is, the younger the proband, the higher the risks for breast and ovarian cancers in her mother and sisters. Further, there was a tendency for these tumours to occur earlier in life if the proband was diagnosed with breast cancer at an early age. The occurrence of bilateral breast cancer or breast plus ovarian cancer in a proband was a strong predictor of breast and ovarian cancers among female relatives (Table 7). Of the 201 probands with bilateral breast cancer, 31 of their 191 mothers (16%) and 25 of their 186 sisters (13%) developed cancers of the breast or ovary. Of the 20 probands with breast cancer and subsequent ovarian cancer, 8 of their 19 mothers (42%) and 5 of their 23 sisters (22%) also developed one of these malignancies.

**Table 3** Standardized incidence ratios (SIRs) of second malignant neoplasms (SMN) of the breast, ovary and other sites among 2860 women with early-onset breast cancer, 1943–90, and follow-up through 1993

| Site of SMN (ICD-7 code) | Obs | Exp | SIR | 95% CI |
|---|---|---|---|---|
| All malignant neoplasms (140–204) | 268 | 68.9 | 3.9 | 3.4–4.4 |
| Breast[a] (170) | 181 | 24.5 | 7.4 | 6.4–8.6 |
| Ovary (175) | 20 | 3.3 | 6.0 | 3.7–9.2 |
| All other sites combined | 67 | 41.1 | 1.6 | 1.3–2.1 |
| Endometrium (172) | 5 | 1.8 | 2.7 | 0.9–6.4 |
| Cervix uteri (171) | 9 | 6.8 | 1.3 | 0.6–2.5 |
| Buccal cavity and pharynx (140–148) | 1 | 0.7 | 1.5 | 0.0–8.1 |
| Stomach (151) | 2 | 0.7 | 3.0 | 0.3–11 |
| Colon (153) | 5 | 2.3 | 2.2 | 0.7–5.2 |
| Rectum (154) | 3 | 1.1 | 2.8 | 0.6–8.1 |
| Liver (155) | 2 | 0.2 | 9.4 | 1.1–34 |
| Biliary tract (155.1) | 0 | 0.2 | 0.0 | 0.0–18.4 |
| Pancreas (157) | 1 | 0.6 | 1.8 | 0.8–9.9 |
| Larynx (161) | 0 | 0.2 | 0.0 | 0.0–18.4 |
| Lung (162) | 9 | 3.3 | 2.7 | 1.2–5.2 |
| Urinary system (180,181) | 2 | 1.5 | 1.3 | 0.2–4.8 |
| Melanoma of skin (190) | 5 | 4.1 | 1.2 | 0.4–2.8 |
| Non-melanoma of skin (191) | 13 | 8.7 | 1.5 | 0.8–2.6 |
| Brain (193) | 4 | 2.8 | 1.4 | 0.4–3.7 |
| Thyroid (194) | 2 | 0.7 | 3.0 | 0.3–10.8 |
| Lymphatic and haematopoietic tissues (200–204) | 4 | 2.7 | 1.5 | 0.4–3.8 |
| Other and unspecified sites | 0 | 2.7 | 0.0 | 0.0–1.4 |

Obs, number of cases observed; Exp, number of cases expected. [a]Excluding 21 women with synchronous, bilateral breast cancer.

© Cancer Research Campaign 1999

**Table 4** Standardized incidence ratios (SIRs) for cancer in mothers and sisters of 2840 probands with early-onset breast cancer

| Site of cancer (ICD-7 code) | Mothers (*n* = 2670) | | | | Sisters (*n* = 2156) | | | |
|---|---|---|---|---|---|---|---|---|
| | Obs | Exp | SIR | 95% CI | Obs | Exp | SIR | 95% CI |
| All malignant neoplasms (140–204) | 889 | 680.4 | 1.3 | 1.2–1.4 | 196 | 123.9 | 1.6 | 1.4–1.8 |
| Breast (170) | 291 | 147.5 | 2.0 | 1.8–2.2 | 92 | 35.8 | 2.6 | 2.1–3.2 |
| Ovary (175) | 64 | 38.5 | 1.7 | 1.3–2.1 | 12 | 6.5 | 1.9 | 1.0–3.3 |
| All other sites combined | 534 | 494.4 | 1.1 | 1.0–1.2 | 92 | 81.6 | 1.1 | 0.9–1.4 |
| Endometrium (172) | 38 | 40.6 | 0.9 | 0.7–1.3 | 6 | 4.4 | 1.4 | 0.5–3.0 |
| Cervix uteri (171) | 84 | 60.0 | 1.4 | 1.2–1.7 | 15 | 13.5 | 1.1 | 0.6–1.8 |
| Buccal cavity and pharynx (140–148) | 9 | 7.6 | 1.2 | 0.5–2.3 | 3 | 1.4 | 2.1 | 0.4–6.1 |
| Stomach (151) | 17 | 17.1 | 1.0 | 0.6–1.6 | 1 | 1.4 | 0.7 | 0.0–3.9 |
| Colon (153) | 67 | 53.8 | 1.2 | 1.0–1.6 | 5 | 4.9 | 1.0 | 0.3–2.4 |
| Rectum (154) | 30 | 27.3 | 1.1 | 0.8–1.6 | 2 | 2.3 | 0.9 | 0.1–3.1 |
| Liver (155) | 4 | 4.3 | 0.9 | 0.3–2.4 | 0 | 0.5 | 0.0 | 0.0–7.4 |
| Biliary tract (155.1) | 8 | 7.9 | 1.0 | 0.4–2.0 | 1 | 0.5 | 2.1 | 0.0–12 |
| Pancreas (157) | 12 | 17.6 | 0.7 | 0.4–1.2 | 1 | 1.3 | 0.8 | 0.0–4.3 |
| Larynx (161) | 4 | 2.2 | 1.8 | 0.5–4.7 | 0 | 0.4 | 0.0 | 0.0–9.2 |
| Lung (162) | 48 | 44.8 | 1.1 | 0.8–1.4 | 12 | 7.0 | 1.7 | 0.9–3.0 |
| Kidney (180) | 19 | 15.9 | 1.2 | 0.7–1.9 | 0 | 1.8 | 0.0 | 0.0–2.0 |
| Bladder (181) | 12 | 17.3 | 0.7 | 0.4–1.2 | 2 | 1.8 | 1.1 | 0.1–4.0 |
| Melanoma of skin (190) | 12 | 14.1 | 0.9 | 0.4–1.5 | 4 | 6.6 | 0.6 | 0.2–1.6 |
| Non-melanoma of skin (191) | 75 | 67.4 | 1.1 | 0.9–1.4 | 12 | 13.1 | 0.9 | 0.5–1.6 |
| Brain (193) | 10 | 17.8 | 0.6 | 0.3–1.0 | 7 | 5.9 | 1.2 | 0.5–2.4 |
| Bone and connective tissue (196, 197) | 2 | 2.9 | 0.7 | 0.1–2.5 | 1 | 1.2 | 0.8 | 0.0–4.6 |
| Non-Hodgkin's lymphoma (200, 202) | 16 | 11.4 | 1.4 | 0.8–2.3 | 3 | 2.3 | 1.3 | 0.3–3.8 |
| Hodgkin's disease (201) | 4 | 2.7 | 1.5 | 0.4–3.8 | 1 | 1.5 | 0.7 | 0.0–3.7 |
| Multiple myeloma (203) | 7 | 6.1 | 1.2 | 0.5–2.4 | 0 | 0.5 | 0.0 | 0.0–7.4 |
| Leukemia (204) | 9 | 12.5 | 0.7 | 0.3–1.4 | 7 | 3.0 | 2.3 | 0.9–4.8 |
| Other and unspecified sites | 47 | 43.1 | 1.1 | 0.8–1.5 | 9 | 6.3 | 1.4 | 0.7–2.7 |

Obs, number observed; Exp, number expected.

**Table 5** Standardized incidence ratios (SIRs) of breast cancer among first-degree female relatives (mothers and sisters) of probands by age of the relative and by age of the proband when her own breast cancer was diagnosed

| Age of proband at diagnosis (years) | Age of female relative (years) | | | | | | | |
|---|---|---|---|---|---|---|---|---|
| | <40 | | 40–59 | | ≥60 | | All ages | |
| | SIR | Obs | SIR | Obs | SIR | Obs | SIR | Obs |
| <30 | 7.1[a] | 10 | 1.8[a] | 17 | 2.2[a] | 15 | 2.3[a] | 42 |
| 30–34 | 4.4[a] | 16 | 2.7[a] | 68 | 1.7[a] | 33 | 2.4[a] | 117 |
| 35–39 | 2.2[a] | 17 | 2.1[a] | 118 | 1.7[a] | 89 | 1.9[a] | 224 |
| All ages | 3.3[a] | 43 | 2.2[a] | 203 | 1.7[a] | 137 | 2.1[a] | 383 |

Obs, number of cases observed. [a]*P* <0.05.

**Table 6** Standardized incidence ratios (SIRs) of breast cancer among first-degree female relatives (mothers and sisters) of probands by age of the relative and by age of the proband when her own breast cancer was diagnosed

| Age of proband at diagnosis (years) | Age of female relative (years) | | | | | | | |
|---|---|---|---|---|---|---|---|---|
| | <40 | | 40–59 | | ≥60 | | All ages | |
| | SIR | Obs | SIR | Obs | SIR | Obs | SIR | Obs |
| <30 | 2.8 | 1 | 3.7[a] | 8 | 0.6 | 1 | 2.3[a] | 10 |
| 30–34 | 7.6[a] | 7 | 3.1[a] | 18 | 1.4 | 7 | 2.7[a] | 32 |
| 35–39 | 1.0 | 2 | 1.4 | 19 | 1.0 | 13 | 1.2 | 34 |
| All ages | 3.1[a] | 10 | 2.1[a] | 45 | 1.0 | 21 | 1.7[a] | 76 |

Obs, number of cases observed. [a]*P*<0.05

Figure 1 shows the cumulative risk of breast or ovarian cancer by the age of the mothers and sisters up to age 75 years, compared with corresponding risk estimates for the general population. Approximately 15% (one in seven) of mothers and sisters of women with early-onset breast cancer were diagnosed with breast or ovarian cancer before age 75 vs 8% (1 in 12) in the general population. The familial risk increased from 14% when the probands were diagnosed at ages 35–39 to 18% when the probands were diagnosed under the age of 35. The cumulative incidence by age 75 reached 24% (one in four) among mothers and sisters of probands with bilateral breast cancer or with breast and subsequent ovarian cance r.

### Male relatives

Among the 2587 fathers of women with early-onset breast cancer (person–years of follow-up 97 000; average 37 years; range, 0.3–51 years), 798 cancers were recorded, with 744.7 expected, yielding an SIR of 1.1 (Table 8). Among the 2354 brothers (person–years of follow-up, 101 000; average 43 years; range

© Cancer Research Campaign 1999

**Table 7** Standardized incidence ratios (SIRs) of cancers of the breast, ovary and other sites combined among first-degree female relatives (mothers and sisters) of 201 and 20 probands with bilateral breast cancer or a second primary cancer of the ovary respectively

| | Probands with cancer of | | | | | | | |
|---|---|---|---|---|---|---|---|---|
| | Bilateral breast[a] (*n* = 201) | | | | Breast and ovary (*n* = 20) | | | |
| Cancer sites in female relatives | Obs | Exp | SIR | 95% CI | Obs | Exp | SIR | 95% CI |
| Mothers | | | | | | | | |
| Breast | 26 | 10.2 | 2.6 | 1.7–3.8 | 3 | 0.9 | 3.2 | 0.7–9.5 |
| Ovary | 5 | 2.7 | 1.9 | 0.6–4.3 | 5 | 0.2 | 20.4 | 6.6–47 |
| Other sites | 31 | 34.8 | 0.9 | 0.6–1.3 | 5 | 3.2 | 1.6 | 0.5–3.6 |
| Sisters | | | | | | | | |
| Breast | 21 | 3.4 | 6.1 | 3.8–9.4 | 5 | 0.6 | 9.2 | 3.0–21 |
| Ovary | 4 | 0.6 | 6.4 | 1.7–16 | 0 | 0.1 | 0.0 | 0.0–37 |
| Other sites[b] | 13 | 7.9 | 1.7 | 0.9–2.8 | 1 | 1.2 | 0.8 | 0.0–4.6 |
| Mothers and sisters | | | | | | | | |
| Breast | 47 | 13.6 | 3.5 | 2.5–4.6 | 8 | 1.5 | 5.4 | 2.3–11 |
| Ovary | 9 | 3.3 | 2.7 | 1.2–5.2 | 5 | 0.3 | 15.5 | 5.0–36 |
| Other sites | 44 | 42.7 | 1.0 | 0.8–1.4 | 6 | 4.4 | 1.4 | 0.3–3.2 |

Obs, number observed; Exp, number expected; [a]Includes 21 women with synchronous, bilateral early-onset breast cancer and one pair of sisters both with bilateral breast cancer, of whom only the sister first diagnosed is regarded as the proband; [b] Includes three cases of cervical cancer (1.3 expected) two cases of endometrial cancer (0.4 expected) and four cases of skin cancer (1.9 expected)

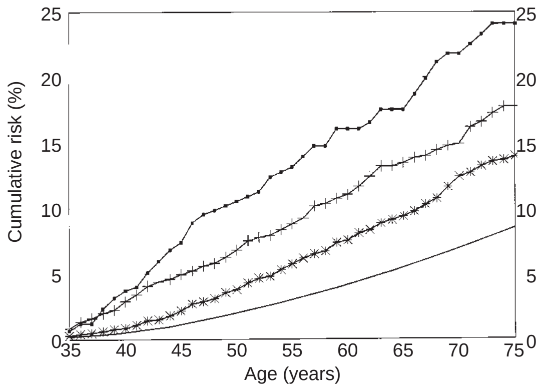

**Figure 1** Cumulative risk for breast and ovarian cancer in mothers and sisters of probands who developed either bilateral breast cancer or breast plus ovarian cancer (⊡); who developed breast cancer before age 35 (+); and who developed breast cancer when 35–39 years of age(⊠); compared with the expected percentage in the general population (–)

>0–51 years), 107 cancers were seen, with 89.6 expected, yielding a SIR of 1.2. Most prominent were the risks for breast cancer among the fathers (SIR 2.5; 95% CI 0.5–7.4) and brothers (29; 7.7–74), although based on small numbers. Significantly increased risks were also seen for cancer of the buccal cavity and pharynx (SIR 1.4), non-melanoma skin cancer (1.2), and non-Hodgkin's lymphoma (1.6) in fathers.

A total of 2662 sons of women with early-onset breast cancer were found, representing approximately 58 000 person–years. Their average age in 1993 was only 21.8 years. Nine cancers were observed, with 13.4 expected, yielding an SIR of 0.7 (95% CI 0.3–1.3). Three cases of malignant melanoma were seen compared with 0.5 expected (SIR 6.4; 1.3–19). There were no cases of male breast cancer, leukaemia, sarcoma or brain cancer.

### Childhood tumours

A total of 127 000 person–years of follow-up were accrued during childhood, i.e. ages 0–14 in the combined group of daughters and sons (73 500 person–years) and sisters and brothers (53 500 person–years). Overall, 26 cancers were observed, with 16.8 expected (SIR 1.6; 95% CI 1.0–2.3). Of these, nine were leukaemias vs 5.5 expected (1.6; 0.8–3.1), six were brain tumours vs 4.9 expected (1.2; 0.5–2.7), three were non-Hodgkin's lymphomas vs 1.0 expected (2.9; 0.6–8.5), two were Hodgkin's disease vs 0.5 expected (4.3; 0.5–16), and two were cancers of the buccal cavity and pharynx vs 0.2 expected (13; 1.5–48). The remaining four cases consisted of a mediastinal tumour, liver cancer (not specified as primary), cutaneous melanoma, and cancer of unspecified site. There were no sarcomas of bone or connective tissue.

### DISCUSSION

Women who develop breast cancer before the age of 40 are at very high risk of developing a subsequent breast or ovarian cancer, with the overall relative risk reaching sevenfold in our study. This substantial excess risk is probably due to genetic predisposition, although radiotherapy given to young patients may contribute to the occurrence of contralateral breast cancer (Boice et al, 1992; Storm et al, 1992). For mothers and sisters of women with early-onset breast cancer, the overall relative risk for breast and ovarian cancer was twofold, and the cumulative risk at 75 years of age was 15% in comparison with 8% expected in the general female population of Denmark. Consistent with other studies of families with breast and ovarian cancers (Claus et al, 1990; Slattery and Kerber, 1993; Teare et al, 1994; Peto et al, 1996), the earlier the age at onset of breast cancer in the proband, the greater the familial risk of breast or ovarian cancer. The lifetime risk rose further, to about 24%, when the proband had bilateral breast cancer or subsequent

*© Cancer Research Campaign 1999*

**Table 8** Standardized incidence ratios (SIRs) for cancer in fathers and brothers of 2840 probands with early-onset breast cancer

| Site of cancer (ICD-7 code) | Father (*n* = 2587) | | | | Brothers (*n* = 2354) | | | |
|---|---|---|---|---|---|---|---|---|
| | Obs | Exp | SIR | 95% CI | Obs | Exp | SIR | 95% CI |
| All malignant neoplasms (140–204) | 798 | 744.7 | 1.1 | 1.0–1.2 | 107 | 89.6 | 1.2 | 1.0–1.4 |
| Breast (170) | 3 | 1.2 | 2.5 | 0.5–7.4 | 4 | 0.1 | 28.9 | 7.7–74 |
| Prostate (177) | 76 | 76.9 | 1.0 | 0.8–1.2 | 2 | 2.1 | 1.0 | 0.1–3.5 |
| Testis (178) | 9 | 5.6 | 1.6 | 0.7–3.0 | 5 | 8.7 | 0.6 | 0.2–1.3 |
| Buccal cavity and pharynx (140–148) | 31 | 21.9 | 1.4 | 1.0–2.0 | 6 | 3.6 | 1.7 | 0.6–3.7 |
| Stomach (151) | 41 | 40.1 | 1.0 | 0.7–1.4 | 3 | 2.3 | 1.3 | 0.3–3.8 |
| Colon (153) | 50 | 52.6 | 1.0 | 0.7–1.3 | 6 | 4.3 | 1.4 | 0.5–3.1 |
| Rectum (154) | 41 | 43.3 | 1.0 | 0.7–1.3 | 2 | 2.0 | 0.7 | 0.1–2.4 |
| Liver (155) | 5 | 7.9 | 0.6 | 0.2–1.5 | 0 | 0.7 | 0.0 | 0.0–5.3 |
| Biliary tract (155.1) | 5 | 5.3 | 1.0 | 0.3–2.2 | 0 | 0.4 | 0.0 | 0.0–9.2 |
| Pancreas (157) | 30 | 23.4 | 1.3 | 0.9–1.8 | 2 | 1.7 | 1.2 | 0.1–4.3 |
| Larynx (161) | 13 | 12.2 | 1.1 | 0.6–1.8 | 1 | 1.5 | 0.7 | 0.0–3.7 |
| Lung (162) | 149 | 146.2 | 1.0 | 0.9–1.2 | 9 | 9.9 | 0.9 | 0.4–1.7 |
| Kidney (180) | 27 | 23.6 | 1.1 | 0.8–1.7 | 4 | 2.8 | 1.5 | 0.4–3.7 |
| Urinary bladder (181) | 75 | 65.0 | 1.2 | 0.9–1.5 | 4 | 5.3 | 0.8 | 0.2–1.9 |
| Melanoma of skin (190) | 9 | 9.8 | 0.9 | 0.4–1.7 | 8 | 4.4 | 1.8 | 0.8–3.6 |
| Non-melanoma of skin (191) | 122 | 91.9 | 1.3 | 1.1–1.6 | 18 | 12.9 | 1.4 | 0.8–2.2 |
| Brain (193) | 16 | 17.6 | 0.9 | 0.5–1.5 | 8 | 6.5 | 1.2 | 0.5–2.4 |
| Bone and connective tissue (196, 197) | 2 | 3.1 | 0.6 | 0.1–2.3 | 3 | 1.7 | 1.8 | 0.4–5.2 |
| Non-Hodgkin's lymphoma (200, 202) | 22 | 14.1 | 1.6 | 1.0–2.4 | 4 | 3.5 | 1.1 | 0.3–2.9 |
| Hodgkin's disease (201) | 3 | 4.2 | 0.7 | 0.1–2.8 | 6 | 2.6 | 2.3 | 0.8–5.0 |
| Multiple myeloma (203) | 4 | 8.5 | 0.5 | 0.1–1.2 | 0 | 0.7 | 0.0 | 0.0–5.3 |
| Leukemia (204) | 19 | 19.9 | 1.0 | 0.6–1.5 | 7 | 4.4 | 1.6 | 0.6–3.3 |
| Other and unspecified sites | 46 | 50.4 | 0.9 | 0.7–1.2 | 5 | 7.5 | 0.7 | 0.2–1.6 |

Obs, number observed; Exp, number expected.

ovarian cancer. These results cannot readily be explained by recall or selection bias, because all family members during a 50-year period in the entire country of Denmark were identified from national population and church registers, and cancer occurrence was determined from national cancer registry files.

Germline mutations of the *BRCA1* susceptibility gene are estimated to account for about 3% of all breast cancers and 4–5% of all ovarian cancers (Ford et al, 1995; Whittemore et al, 1997). If all of the excess breast and ovarian cancers in the mothers of women who developed breast cancer under the age of 40 in our study were associated with inherited mutations, 6.3% of the mothers (169 of 2670) would have transmitted a susceptibility gene to their daughters. Taking into account that some women with family histories may have mutations in genes other than *BRCA1*, this estimate is consistent with the approximately 6–13% prevalence of *BRCA1* mutations found in sporadic cases of breast cancer in women under the age of 35 (FitzGerald et al, 1996; Langston et al, 1996; Struewing et al, 1996; Malone et al, 1998), with about half of the mutations inherited from mothers and half from fathers.

Population studies in Iceland and the United Kingdom have also reported familial aggregation of incident or fatal cases of breast and ovarian cancers in relatives of young breast cancer patients, with relative risks similar to those we observed (Anderson et al, 1992; Tulinius et al, 1992*b*; Teare et al, 1994; Peto et al, 1996). Some population and family studies of breast cancer have also suggested excess risks for cancers of the colon (Goldgar et al, 1994), endometrium (Anderson et al, 1992; Tulinius et al, 1992*a*; Peto et al, 1996), and prostate (Tulinius et al, 1992*b*; Anderson and Badzich, 1993; Sellers et al, 1994; Tulinius et al, 1994; Struewing et al, 1997), but our findings are consistent with studies (Schildkraut et al, 1989; Negri et al, 1997) showing no elevated risk for these tumours except possibly colon cancer in mothers. The excess risk for cervical cancer we observed in mothers did not extend to the sisters or daughters and probably represents a chance occurrence. A significant increase in the risk for lung cancer among the probands may be related to radiotherapy (Inskip et al, 1994) rather than to inherited factors, although a slight non-significant increase in the risk for lung cancer was noted in mothers and sisters suggesting familial patterns of tobacco use.

Despite its rarity, the risk of male breast cancer was significantly elevated in accord with family studies indicating a genetic susceptibility to male and female breast cancer, due especially to germline mutations of *BRCA2* (Stratton and Wooster, 1996). The gene for Cowden's disease has recently been identified as *PTEN* (Li et al, 1997), and may contribute to familial occurrences of breast cancer as well as thyroid cancer.

In Li–Fraumeni syndrome, mutations in the *p53* gene are responsible for the familial constellation of early-onset breast cancers and other tumours of young people, notably sarcomas of soft tissue and bone, acute leukaemia, brain tumours and adrenocortical neoplasms (Li et al, 1988; Garber et al, 1991; Varley et al, 1997). In a previous population-based incidence study of cancer

 © Cancer Research Campaign 1999

risks among the parents of all children with cancer in Denmark, we were able to detect a familial association between early-onset breast cancer and childhood sarcoma (Olsen et al, 1995). The current approach covered nearly 50 years of breast cancer incidence in Denmark, but still had insufficient statistical power to thoroughly assess the constellation of tumours associated with rare genetic conditions such as Li–Fraumeni syndrome.

Thus, our population-based survey of cancer incidence among close relatives of women with early-onset breast cancer in Denmark suggests that susceptibility genes to breast cancer contribute to only a small percentage of non-mammary malignancies other than those of the ovary.

### REFERENCES

Amos CI and Struewing JP (1993) Genetic epidemiology of epithelial ovarian cancer. *Cancer* **71** (suppl. 2): 566–572

Anderson DE and Badzioch MD (1993) Familial breast cancer risks: effects of prostate and other cancers. *Cancer* **72**: 114–119

Anderson KE, Easton DF, Matthews FE and Peto J (1992) Cancer mortality in the first degree relatives of young breast cancer patients. *Br J Cancer* **66**: 599–602

Bebb G, Glickman B, Gelman K and Gatti R (1997) ‘At risk’ for breast cancer (commentary). *Lancet* **349**: 1784–1785

Boice Jr JD, Harvey E, Blettner M, Stovall M and Flannery JT (1992) Cancer in the contralateral breast after radiotherapy for breast cancer. *N Engl J Med* **326**: 781–785

Claus EB, Risch NJ and Thompson WD (1990) Age at onset as an indicator of familial risk of breast cancer. *Am J Epidemiol* **131**: 961–972

Claus EB, Risch NJ and Thompson WD (1991) Genetic analysis of breast cancer in the Cancer and Steroid Hormone Study. *Am J Hum Genet* **48**: 232–242

Crook T, Crossland S, Crompton MR, Osin P and Gusterson BA (1997) *p53* mutations in *BRCA1*-associated familial breast cancer. *Lancet* **350**: 638–639

Danish Board of Health (1997) Cancer incidence in Denmark 1994. In *Health Statistics*, vol. 7. Danish Board of Health: Copenhagen

Eby N, Chang-Claude J and Bishop DT (1994) Familial risk and genetic susceptibility for breast cancer. *Cancer Causes Control* **4**: 458–470

FitzGerald MG, MacDonald DJ, Krainer M, Hoover I, O’Neil E, Unsal H, Silva-Arrieto S, Finkelstein DM, Beer-Romero P, Englert C, Sgroi DC, Smith BL, Younger JW, Garber JE, Duda RB, Mayzel KA, Isselbacher KJ, Friend SH and Haber DA (1996) Germ-line *BRCA1* mutations in Jewish and non-Jewish women with early-onset breast cancer. *N Engl J Med* **334**: 143–149

FitzGerald MG, Bean JM, Hegde SR, Unsal H, MacDonald DJ, Harkin DP, Finkelstein DM, Isselbacher KJ and Haber DA (1997) Heterozygous ATM mutations do not contribute to early onset of breast cancer. *Nature Genet* **15**: 307–310

Ford D, Easton DF and Peto J (1995) Estimates of the gene frequency of *BRCA1* and its contribution to breast and ovarian cancer incidence. *Am J Hum Genet* **57**: 1457–1462

Garber JE, Goldstein AM, Kantor AF, Dreyfus MG, Fraumeni Jr JF and Li FP (1991) Follow-up study of twenty-four families with Li–Fraumeni syndrome. *Cancer Res* **51**: 6094–6097

Goldgar DE, Easton DF, Cannon-Albright LA and Skolnick MH (1994) Systematic population-based assessment of cancer risk in first-degree relatives of cancer probands. *J Natl Cancer Inst* **86**: 1600–1608

Gudmundsson J, Johannesdottir G, Arason A, Bergthorsson JT, Ingvarsson S, Egilsson V and Barkardottir RB (1996) Frequent occurrence of *BRCA2* linkage in Icelandic breast cancer families and segregation of a common *BRCA2* haplotype. *Am J Hum Genet* **58**: 749–756

Inskip PD, Stovall M and Flannery JT (1994) Lung cancer risk and radiation dose among women treated for breast cancer. *J Natl Cancer Inst* **86**: 983–988

Krainer M, Silva-Arrieta S, FitzGerald MG, Shimada A, Ishioka C, Kanamaru R, MacDonald DJ, Unsal H, Finkelstein DM, Bowcock A, Isselbacher KJ and Haber DA (1997) Differential contributions of *BRCA1* and *BRCA2* to early-onset breast cancer. *N Engl J Med* **336**: 1416–1421

Langston AA, Malone KE, Thompson JD, Daling JR and Ostrander EA (1996) *BRCA1* mutations in a population-based sample of young women with breast cancer. *N Engl J Med* **334**: 137–142

Li FP, Fraumeni Jr JF, Mulvihill JJ, Blattner WA, Dreyfus MG, Tucker MA and Miller RW (1988) A cancer family syndrome in twenty-four kindreds. *Cancer Res* **48**: 5358–5362

Li J, Yen C, Liaw D, Podsypanina K, Bose S, Wang SI, Puc J, Miliaresis C, Rodgers L, McCombie R, Bigner SH, Giovanella BC, Ittmann M, Tycko B, Hibshoosh H, Wigler MH and Parsons R (1997) *PTEN* a putative protein tyrosine phosphatase gene mutated in human brain, breast, and prostate cancer. *Science* **275**: 1943–1947

Malone KE, Daling JR, Thompson JD, O’Brien CA, Francisco LV and Ostrander EA (1998) *BRCA1* mutations and breast cancer in the general population. *JAMA* **279**: 922–929

Negri E, Braga C, Vecchia CL, Franceschi S and Parazzini F (1997) Family history of cancer and risk of breast cancer. *Int J Cancer* **72**: 735–738

Newman B, Millikan RC and King M-C (1997) Genetic epidemiology of breast and ovarian cancers. *Epidemiol Rev* **19**: 69–79

Olsen JH, Boice Jr JD, Seersholm N, Bautz A and Fraumeni Jr JF (1995) Cancer in parents of children with cancer. *N Engl J Med* **333**: 1594–1599

Peto J, Easton DF, Matthews FE, Ford D and Swerdlow AJ (1996) Cancer mortality in relatives of women with breast cancer: the OPCS study. *Int J Cancer* **65**: 275–283

Pharoah PD, Day NE, Duffy S, Easton DF and Ponder BA (1997) Family history and the risk of breast cancer: a systematic review and meta-analysis. *Int J Cancer* **71**: 800–809

Rebbeck TR, Couch KJ, Kant J, Calzone K, DeShano M, Peng Y, Chen K, Garber JE and Weber BL (1996) Genetic heterogenicity in hereditary breast cancer: role of *BRCA1* and *BRCA2*. *Am J Hum Genet* **59**: 547–553

Rothman KJ and Boice Jr JD (1979) *Epidemiologic Analysis with a Programmable Calculator*. DHHS publication no. (NIH) 79-1649. Government Printing Office: Washington, DC

Schildkraut JM, Risch N and Thompson WD (1989) Evaluating genetic association among ovarian, breast, and endometrial cancer: evidence for a breast/ovarian cancer relationship. *Am J Hum Genet* **45**: 521–529

Sellers TA, Potter JD, Rich SS, Drinkard CR, Bostick RM, Kushi LH, Zheng W and Folsom AR (1994) Familial clustering of breast and prostate cancers and risk of postmenopausal breast cancer. *J Natl Cancer Inst* **86**: 1860–1865

Slattery ML and Kerber RA (1993) A comprehensive evaluation of family history and breast cancer risk: the Utah Population Database. *J Am Med Assoc* **270**: 1563–1568

Storm HH, Andersson M, Boice Jr JD, Blettner M, Stovall M, Mouridsen HT, Dombernowsky P, Roce C, Jacobsen A and Pedersen M (1992) Adjuvant radiotherapy and risk of contralateral breast cancer. *J Natl Cancer Inst* **84**: 1245–1250

Stratton MR and Wooster R (1996) Hereditary predisposition to breast cancer. *Curr Opinions Genet Dev* **6**: 93–97

Struewing JP, Tarone RE, Li F, Brody LC and Boice Jr JD (1996) *BRCA1* mutations in young women with breast cancer (letter). *Lancet* **347**: 1493

Struewing JP, Hartge P, Wacholder S, Baker SM, Berlin M, McAdams M, Timmerman MM, Brody LC and Tucker MA (1997) The risk of cancer associated with specific mutations of *BRCA1* and *BRCA2* among Ashkenazi Jews. *N Engl J Med* **336**: 1401–1408

Teare MD, Wallace SA, Harris M, Howell A and Birch JM (1994) Cancer experience in the relatives of an unselected series of breast cancer patients. *Br J Cancer* **70**: 102–111

Tulinius H, Egilsson V, Olafsdottir GH and Sigvaldson (1992*a*) Risk of prostate, ovarian, and endometrial cancer among relatives of women with breast cancer. *Br Med J* **305**: 855–857

Tulinius H, Sigvaldsson H, Olafsdottir G and Tryggvadottir L (1992*b*) Epidemiology of breast cancer in families in Iceland. *J Med Genet* **29**: 158–164

Tulinius H, Olafsdottir GH, Sigvaldason H, Tryggvadottir L and Bjarnadottir K (1994) Neoplastic diseases in families of breast cancer patients. *J Med Genet* **31**: 618–621

Varley JM, Evans DG and Birch JM (1997) Li–Fraumeni syndrome – a molecular and clinical review. *Br J Cancer* **76**: 1–14

Whittemore AS, Gong G and Itnyre J (1997) Prevalence and contribution of *BRCA1* mutations in breast cancer and ovarian cancer: results from three US population-based case–control studies of ovarian cancer. *Am J Hum Genet* **60**: 496–504

World Health Organization (1957) *Manual of the International Statistical Classification of Diseases, Injuries, and Causes of Death*, 7th rev. World Health Organization: Geneva

© Cancer Research Campaign 1999
